# Ecological Effects of *Solanum rostratum* Invasion on the Diversity and Functional Traits of Native Plant Communities

**DOI:** 10.1002/ece3.72910

**Published:** 2026-01-11

**Authors:** Lijun Hu, Lamei Jiang, Juan Qiu, Amanula Yimingniyazi

**Affiliations:** ^1^ College of Resources and Environment Xinjiang Agricultural University Urumqi China; ^2^ Xinjiang Key Laboratory of Ecological Adaptation and Evolution of Extreme Environment Biology, College of Life Sciences Xinjiang Agricultural University Urumqi China

**Keywords:** degree of invasion, functional diversity, invasive plants, species diversity

## Abstract

Severe invasion by invasive plants can reduce the diversity of native plants, thereby limiting the functional diversity of ecosystems and threatening their stability in the invaded areas. In this study, the invasion area of *Solanum rostratum* in Urumqi, Xinjiang, China, was analyzed to determine the impact of different degrees of invasion on the species and functional diversity of plant communities in desert steppe and the ecological effects and driving mechanisms of invasion. Community‐weighted mean trait values of invasive and coexisting local plants were also calculated to explore changes in species diversity and functional diversity at different degrees of invasion. *S. rostratum* invasion was associated with significant differences in soil nutrient patterns and exhibited phase‐specific disturbance characteristics. Compared with the non‐invaded quadrats, low and moderate invasion levels increased local community diversity and stability (*p* < 0.05), whereas high invasion significantly reduced local community diversity (*p* < 0.05). The community stability index dropped from 2.29 (light invasion) to 1.23 (severe invasion), representing a decrease of 46.21%. The invasibility index rose from 0.29 to 0.51, representing an increase of 73.86%. The *S. rostratum* invasion index increased from 0.64 to 1.95, representing an increase of 50.27%. Thus, plant species diversity was the primary factor affecting the stability and invasiveness of native plant communities. The weighted average character value of invasive plants was higher than that of native plants; the functional difference was < 0 for the low‐ and medium‐invasion quadrats and > 0 for the high‐invasion quadrat. *S. rostratum* invasion restructured soil nutrient regimes. The competitive strategy of *S. rostratum* is to promote and restrict local plant growth under low and high invasion levels, respectively, leading to a sharp decline in biodiversity and ecosystem imbalance. The results indicate that timely control measures should be implemented upon the initial invasion to prevent further spread and maintain ecological security, sustainable agriculture, and animal husbandry development.

## Graphical abstract

The invasion of *Solanum rostratum* exerts stage‐dependent effects on native plant communities. The study found that at low invasion levels, it temporarily enhances native plant diversity and community stability through resource complementarity; whereas at high invasion levels, it shifts to strong competitive inhibition, leading to a sharp decline in species diversity and ecosystem imbalance. This process is accompanied by a functional trait shift from “convergence to divergence”: during early invasion, functional traits converge with those of native plants, while later stages achieve replacement through superior traits such as greater plant height and larger leaf area. Moreover, the decline in species diversity is not synchronized with changes in functional diversity, indicating a loss of functional redundancy under high invasion intensity. These findings support three core hypotheses—the “diversity‐stability threshold,” “functional trait replacement,” and “loss of functional redundancy”—and underscore the importance of implementing control measures during early invasion stages and maintaining native species diversity to resist invasion and ensure ecological security.

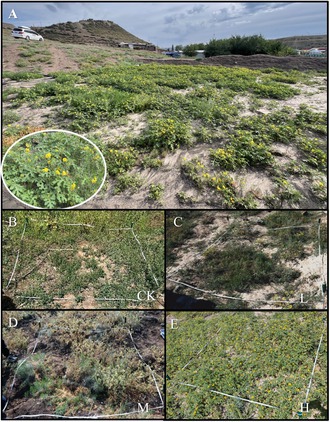

## 1 Introduction

The continuous expansion of invasive plants has a significant negative impact on ecosystem community structures and ecological functions and may lead to long‐term degradation of the service functions of the entire ecosystem (Qian and Shenhua 2022). In recent years, research on the species diversity and functional traits of native plant communities has become a hot topic in ecology (Chen et al. 2023). The loss of species diversity in native plant communities often leads to the degradation of key ecosystem functions. In these communities, species diversity usually presents as a model of nonlinear responses and spatial gradient differentiation (Peng et al. 2019). This study assessed the effects of *Solanum rostratum* invasion on native plant diversity and functional traits across invasion gradients and identified the ecological drivers underlying these patterns. Analysis of plant functional traits can provide more accurate data on the gradual impact of invasive plants on ecosystem functions (Hussain et al. 2021; Xiao et al. 2023) and help identify early warning signals of ecosystem degradation, thereby providing a basis for formulating targeted ecological restoration schemes.

Plant invasion poses a major threat to ecosystems; the diversity‐stability threshold serves as the gold standard for invasion management. Maintaining native plant diversity above this threshold is the most cost‐effective and sustainable strategy for resisting invasion. Plant invasion success is intrinsically linked to functional trait replacement and functional redundancy loss in native plant communities. Given this understanding, corresponding strategies can be developed for different stages of invasion. This strategy integrates three foundational hypotheses: diversity‐stability threshold hypothesis (Liang et al. 2025); functional trait replacement hypothesis (Bjorkman et al. 2018; Wang, Jiang, et al. 2018; Wang, Zhou, et al. 2018; Xiao et al. 2023); and functional redundancy loss hypothesis (Hatton et al. 2024). Collectively, these hypotheses systematically elucidate the mechanisms used by invasive plants to disrupt the diversity and functional trait composition of native plant communities. The diversity‐stability threshold hypothesis states that when the invasion degree exceeds the critical threshold, local community stability and species diversity will show nonlinear attenuation (Liang et al. 2025). The functional trait replacement hypothesis holds that invasive plants have significantly higher functional trait values than native plants, and changes occur in the functional structure of the community through competitive exclusion and trait substitution (Bjorkman et al. 2018). The functional redundancy loss hypothesis states that functional diversity may increase temporarily due to the substitution of dominant species in the early stage of invasion; however, as the invasion intensifies, the extinction of local species leads to the collapse of functional redundancy and a decrease in the anti‐interference ability of the community (Hatton et al. 2024).

Based on the above assumptions, this study proposes the following specific characteristics of invasion. An invasion intensity threshold exists, below which invasion may temporarily increase diversity and stability through resource complementarity but beyond which diversity and stability decline nonlinearly. The functional trait values (such as plant height and leaf area) of the invasive plant will be significantly higher than those of native plants. Moreover, as invasion intensifies, the difference (functional divergence index) will shift from negative (functional convergence) to positive (functional divergence), indicating the occurrence of trait displacement. During the early stages of invasion, functional diversity may temporarily increase due to the addition of new species. However, under high invasion pressure, the loss of native species may lead to a collapse of functional redundancy, resulting in complex changes in functional diversity that become decoupled from the patterns of species diversity change.

Through field observations, we found that *S. rostratum* is widely distributed in oases, grasslands, desert farmlands, and roadside areas where human activities are frequent and can expand over a large area in a short time. The comparative analysis showed that in the invasion sample plot of *S. rostratum* , the local plant community characteristics, such as plant height, coverage, plant number, species number, and leaf area, were significantly different from those in the control plot in Urumqi, Xinjiang. Of note, solanine and other toxic substances are present in *S. rostratum* , which can cause gastrointestinal bleeding and even death in cattle and sheep, thereby threatening local biodiversity and causing direct economic losses to animal husbandry (Eminniyaz 2015).

*S. rostratum* is an annual invasive weed of the Solanaceae family that is native to North America and has strong adaptation, diffusion, and reproductive abilities (Ozuzu et al. 2024; Yu et al. 2024). It was first recorded in Hong Kong in 1895 (Wang, Gao, et al. 2020; Wang, Wei, et al. 2020) and has since spread to 11 provinces and cities, including Xinjiang, Inner Mongolia, Gansu, Ningxia, Beijing, and Hebei. In Xinjiang, *S. rostratum* has spread to Urumqi, Changji, Turpan, and other regions, and its vertical distribution ranges from −54 to 1200 m above sea level. Domestic analyses of *S. rostratum* have primarily focused on the adaptive mechanisms underlying its rapid expansion (Yu et al. 2024) and the basis for its seed dormancy characteristics (Zhang et al. 2011). Researchers have also investigated the species richness and evenness of local plants in areas affected by the *S. rostratum* invasion. However, different degrees of *S. rostratum* invasion and their effects on the species diversity index, community stability, community invasiveness, or functional diversity of native plant communities have not been widely investigated.

This study used *S. rostratum* as the research object and selected the Shuimogou area in Urumqi, Xinjiang, China, as the research area. This is a typical distribution area of *S. rostratum* invasion, and it exhibits an obvious invasion degree gradient and strong representativeness of community types. This study investigated the ecological impacts of *S. rostratum* invasion at different intensity levels, including a no‐invasion control plot, on native plant communities. This study assessed changes in species diversity, community stability characteristics, community invasibility differences, and responses of native plant functional diversity. Our findings will provide a scientific basis for developing ecological control strategies for *S. rostratum* and conserving native plant biodiversity. The effects of *S. rostratum* on plant communities across varying invasion levels were analyzed using one‐way ANOVA, Pearson correlation analysis, path analysis and null model analyses. The scientific questions addressed are as follows: (1) Do nightshade species impact the diversity of native plant communities under different degrees of invasion? (2) What similarities and differences in functional traits occur between *S. rostratum* and native plants under different degrees of invasion? (3) How does *S. rostratum* occupy the niches of local desert steppe plants for successful invasion?

## 2 Materials and Methods

### 2.1 Overview of the Study Area

This study was conducted in a saline desert habitat located in Shuimogou District (43.82° N, 87.77° E), Ürümqi City, Xinjiang, with an elevation of 934.0 m. The region experiences a temperate continental climate, characterized by an annual average temperature of 8°C, with extreme monthly averages reaching 34°C in July and −12°C in January. The mean annual precipitation is approximately 300 mm, the annual evaporation reaches 2500 mm, and there are approximately 2808 annual total sunshine hours. The surveyed area was a typical herbaceous weed community, with *S. rostratum* as the sole invasive species. Co‐occurring native plants included *Peganum harmala* , *Portulaca oleracea* , *Artemisia argyi*, *Onopordum acanthium* , and *Polygonum aviculare* . No other invasive plants were detected during the study period, ensuring an independent assessment of the invasive impact of *S. rostratum* . The soil type in this area has been identified as brown calcic soil, and its habitat has been significantly influenced by long‐term grazing activities. The alien invasive species *S. rostratum* has successfully invaded the study area; however, targeted control and management measures have not been implemented.

### 2.2 Sample Plot Setting

The grading method described by Xiao et al. (2023) was applied to the sampling area to evaluate the degree of *S. rostratum* invasion based on its population coverage. Invasion degree was then divided into four levels: no invasion (0%, control [CK]), low‐invasion plot (< 35%, *L*), medium‐invasion plot (35%–75%, *M*), and high‐invasion plot (> 75%, *H*). Six repeated quadrats were set for each invasion degree, and the quadrat size was 2 × 2 m based on the minimum area method. All sampled quadrats were located within the same plant community type.

### 2.3 Plant Community Survey

A plant community survey was performed in September 2024 to identify all herbaceous plant species in each quadrat. The species names were recorded, and the population characteristics and functional traits were measured. For the population characteristics, the number of plants (individuals) per species, average plant height, coverage of each species (percentage of vertical projection area of the aboveground parts in the quadrat area, calculated manually), aboveground biomass (measurements were conducted by collecting entire plants in the field and weighing their fresh mass), and community coverage of the quadrat were measured. Three healthy individuals of each species were selected to determine their plant functional traits (plant height), and three mature and complete leaves were randomly collected from each individual to determine the leaf functional traits (leaf length, width, and area; Table 1). Soil samples from each quadrat were collected using the five‐point sampling method and subsequently analyzed for element content.

**TABLE 1:** Definition ecological significance, and measurement methods for plant functional traits.

Index	Numerical value	Definition	Ecological significance	Determination method
Plant height	Specific height in centimeters (cm)	Distance from the neck of plant root to the top of main stem	It reflects the key strategy and capability of a plant in competing for light resources.	Take measurements with a ruler that has a 1 cm precision.
Leaf length	Specific height in centimeters (cm)	Maximum value of leaf midrib	They collectively determine the size and morphology of the leaf, serving as the fundamental structural units for light acquisition and photosynthesis.	Take measurements with a ruler that has a 0.1 cm precision.
Leaf width	Specific height in centimeters (cm)	Maximum value perpendicular to blade midrib		Take measurements with a ruler that has a 0.1 cm precision.
Blade thickness	Specific height in centimeters (cm)	Distance from upper epidermis to lower epidermis of leaves	It embodies the trade‐offs among photosynthetic efficiency, stress resistance (e.g., to drought, high light, pests, and diseases), and resource investment strategy.	Measure using a 0.1 mm‐precision Vernier caliper.
Leaf area	Specific area in square centimeter (cm^2^)	Blade size	It directly quantifies the total surface area for photosynthesis, transpiration, and gas exchange, acting as a key interface metric connecting the plant to the atmospheric environment.	Perform measurements via the FS‐Leaf1000 imaging analyzer (Shijiazhuang Fansheng Technology Co. Ltd.).

Functional traits were determined based on the methods of Xiao et al. (2023), and we measured five key functional traits closely related to plant growth competitiveness and ecological fitness. For plant height we measured from the base to the apical meristem using a ruler with 0.1 cm precision. For leaf morphology, we measured the length and width using a 0.1 cm precision ruler. Leaf thickness was measured at the midrib region with a digital caliper (0.01 mm precision). Aboveground biomass was measured using an electronic balance with a precision of 0.1 g. Leaf area was quantified digitally using an FS‐leaf1000 leaf image analyzer (Farsight Technology, Shijiazhuang).

### 2.4 Hypothesis Testing Framework

This study is guided by three core theoretical hypotheses to systematically examine the ecological impacts of *S. rostratum* invasion on native plant communities. The data analysis not only includes conventional descriptions of community characteristics but also focuses on directly verifying the predictions of each hypothesis through the following framework:

Diversity‐Stability Threshold Hypothesis Testing Method: The invasion intensity index of *S. rostratum* (III) was used as a proxy indicator of invasion intensity. Species richness (*S*), Shannon‐Wiener index (*H*′), and Simpson index (*D*) were used as indicators of species diversity. The community stability index (ICV) and community invasibility index (CII) were used to characterize stability and invasibility, respectively.

One‐way ANOVA was conducted to analyze the non‐linear relationships between invasion intensity (III) and species diversity indices (*S*, *H*′), and the community stability index (ICV) was used to identify the presence of significant inflection points (i.e., thresholds). Pearson correlation analysis was performed to verify the relationships between species diversity indices (*S*, *H*′, *D*, *J*, *F*) and ICV (positive correlation) and CII (negative correlation). Subsequently, the direct and indirect contributions of various diversity indicators to ICV and CII were quantified through path analysis.

Functional Trait Replacement Hypothesis: The community‐weighted mean (CWM) trait values of *S. rostratum* were calculated and coexisting native plants were identified to determine key functional traits. The functional dissimilarity index (FDj) between the invasive and native species was then calculated.

One‐way ANOVA was used to compare the differences in CWM values between the invasive and native plants under different invasion intensities. Moreover, it was used to compare the FDj values under different invasion intensities (*L*, *M*, and *H*) and conduct post hoc tests to determine the specific invasion stage at which FDj shifts from negative to positive values.

Functional Redundancy Loss Hypothesis Indicator Selection: Multiple functional diversity indices were calculated: functional evenness (FEve), functional divergence (FDiv), functional richness (FRic), and Rao's Q (FDQ). One‐way ANOVA was used to compare the differences in various functional diversity indices under different invasion intensities, with a particular focus on whether the functional diversity indices (e.g., FRic) exhibited opposite trends relative to the species diversity indices (e.g., *S* and *H*′) under high invasion intensity (*H*). A null structural model analysis was conducted to investigate whether plant invasion has led to the loss of functional redundancy in native plant communities.

### 2.5 Data Analysis

We calculated the following indices using the formulas detailed in Table 2: plant diversity indices, impact degree indices, community stability indices, invasibility indices, plant competitive advantage indices, invasion intensity indices, and functional trait divergence indices between invasive and native plants ( *S. rostratum* ) and plant functional diversity indices.

**TABLE 2:** Calculation formulas for indices in the article.

Index	Name	Symbol	Formula	Description	References
α‐Diversity indices	Shannon‐Wiener index	*H*′	$H^{\prime }=-\sum p_{i}\ln p_{i}$	*S*: Species richness in the plot; *p*_*i*_: Proportional abundance of species *i*; *N*: Total abundance.	Shannon and Weaver (1949)
	Simpson's index	*D*	$D=1-\sum p_{i}^{2}$		Simpson (1949)
	Pielou's evenness index	*J*	$J=H^{\prime }/\ln S$		Pielou (1966)
	Margalef's richness index	*F*	$F=\left(S-1\right)/\ln N$		Margalef (1951)
Impact indices	Impact index of *Solanum rostratum* on species richness	DIIS	$\mathrm{DII}=1-\left(\mathrm{SC}/\mathrm{CK}\right)$	SC: Index value in plots invaded by *S. rostratum* ; CK: Index value in non‐invaded control plots.	Wang et al. (2021)
	Impact index of *S. rostratum* on Shannon‐Wiener index	DIIH'			
	Impact index of *S. rostratum* on Pielou's index	DIIJ			
	Impact index of *S. rostratum* on Margalef's index	DIIF			
Community stability and invasibility indices	Community stability index	ICV	ICV=u/δ	*μ*: Mean density of plant species in the plot; *δ*: Standard deviation of species density.	Loreau and De Mazancourt (2013), Shi et al. (2025), Tilman (1999), Wang, Gao, et al. (2020), and Wang, Wei, et al. (2020)
	Community invasibility index	CII	$\mathrm{CII}=1-\left(\text{Maxpi}-\mathrm{pi}\right)$	Maxpi: Maximum relative abundance of *S. rostratum* across all plots; pi: Relative abundance of *S. rostratum* in the plot.	
Competitive advantage and invasion intensity	Competitive advantage index	CAI	CAI=CWMi/CWM	CWMi: Community‐weighted mean trait value of *S. rostratum* ; CWM: Overall community‐weighted mean trait value; pi: Relative abundance of *S. rostratum* in the plot.	Wang, Jiang, et al. (2018) and Wang, Zhou, et al. (2018)
	Invasion intensity index	III	III=pi/Maxpi		
Functional trait differences	Functional divergence index	FDj	FDj = |CWTij − CWTnj|/(CWTij − CWTn)	FDj: Functional divergence indices	Shi et al. (2025) and Villéger et al. (2008)
Functional trait differences	Rao's quadratic entropy index	FDQ	FDQ = ∑∑(dijPiPj)	dij: Functional trait distance between species *i* and *j*; pi, pj: Relative abundances of species *i* and *j*.	Laliberté and Legendre (2010) and Mason et al. (2005)
Functional diversity indices	Functional evenness index	FEve	FEve = ∑min(de × *d*)/∑de	de: Edge lengths in the minimum spanning tree (MST); *d*: Mean edge length.	Laliberté and Legendre (2010) and Vilà et al. (2011)
	Functional divergence index	FDiv	FDiv = ∑pi × di/∑pi × *d*	pi: Relative abundance of species *i*; di: Distance of species *i* to the centroid; *d*: Mean distance.	
	Functional richness index	FRic	FRic = Volume of the convex hull	Calculates the convex hull area (e.g., polygon) or volume (e.g., polyhedron) in functional trait space.	Laliberté and Legendre (2010) and Liu et al. (2021)

The community stability index (ICV = *μ*/*σ*) adopted in this study primarily reflects the structural spatial constancy of communities rather than their temporal resilience or recovery capacity (Tilman 1999). As the research design infers temporal dynamics based on spatial gradients, using ICV as a proxy indicator for community stability is justified (Wang et al. 2021). Future studies could further validate stability mechanisms along the temporal dimension through long‐term monitoring.

The data analysis methodology encompassed five key components: (1) Data preprocessing involving data organization in Excel 2024 and statistical analysis using SPSS 2022 to calculate mean values and standard deviations (mean ± SD) for all indicators; (2) Significance testing was conducted using one‐way ANOVA (*p* < 0.05), followed by Games‐Howell post hoc tests to examine differences across invasion intensities; (3) Pearson correlation analysis to quantify relationships between *S. rostratum* invasion intensity and community diversity, stability and invasibility; (4) Path analysis with direct (*P*) and indirect (*P*′) path coefficients to assess causal relationships; and (5) Null model structural analysis: Conducting data analysis in R to verify whether plant invasion leads to the loss of functional redundancy in native plant communities; (6) Data visualization for null structural model analysis was conducted using R Core Team, 2024, software, while remaining visualizations were processed through Origin 2024.

## 3 Results Analysis

### 3.1 Effects of Different Degrees of *S. rostratum* Invasion on Soil Nutrient Structure

*S. rostratum* invasion significantly restructured soil nutrient profiles across invasion gradients (*p* < 0.05, Table 3). As the invasion intensity increased, the soil water content showed a non‐significant decreasing trend; soil pH, organic carbon (C), and total nitrogen (N) decreased progressively with significant reductions (*p* < 0.05); available phosphorus (P) and nitrate nitrogen (${\mathrm{NO}}_{3}^{-}$) exhibited unimodal dynamics, with an initial decline followed by an increase at higher invasion levels (*p* < 0.05); ammonium nitrogen (${\mathrm{NH}}_{4}^{+}$) displayed an inverse unimodal pattern, with a peak during light invasion and then significant decline under medium and heavy invasion (*p* < 0.05). These stage‐specific changes in soil nutrient patterns, particularly the differentiated responses of nitrogen and phosphorus forms under light and severe invasion, provide an environmental context for subsequent plant functional trait replacement and community stability changes.

**TABLE 3:** Comparison of soil element concentrations across *Solanum rostratum* invasion.

	Wc (%)	pH	C (g/kg)	N (g/kg)	P (g/kg)	${\mathrm{NH}}_{4}^{+}$‐N (g/kg)	${\mathrm{NO}}_{3}^{-}$‐N (g/kg)
CK	4.60 ± 0.71^ns^	8.76 ± 0.15^ab^	17.82 ± 4.27^a^	1.64 ± 0.19^a^	0.60 ± 0.021^ab^	0.038 ± 0.017^a^	0.028 ± 0.035^a^
*L*	4.02 ± 0.35^ns^	9.01 ± 0.30^b^	15.08 ± 2.88^a^	1.16 ± 0.12^b^	0.55 ± 0.031^a^	0.062 ± 0.017^b^	0.0048 ± 0.00082^b^
*M*	3.72 ± 0.22^ns^	8.44 ± 0.16^c^	8.51 ± 2.22^b^	0.59 ± 0.14^c^	0.60 ± 0.064^ab^	0.043 ± 0.011^a^	0.019 ± 0.0096^ab^
*H*	3.67 ± 0.17^ns^	8.49 ± 0.26^bc^	8.64 ± 1.56^b^	0.58 ± 0.070^c^	0.62 ± 0.056^b^	0.046 ± 0.0065^a^	0.017 ± 0.011^ab^

*Note:* Statistical notation: Different superscript letters within the same parameter indicate significant differences (*p* < 0.05); “ns” denotes non‐significant differences (*p* > 0.05).

Abbreviations: C, soil carbon content; CK, non‐invaded control; *H*, heavy invasion; *L*, light invasion; *M*, moderate invasion; N, soil nitrogen conten; ${\mathrm{NH}}_{4}^{+}$‐N, ammonium nitrogen content; ${\mathrm{NO}}_{3}^{-}$‐N, nitrate nitrogen content; P, soil phosphorus content; pH, soil acidity/alkalinity; Wc, soil water content.

### 3.2 Impact of *S. rostratum* on Species Diversity and the Stability of Native Plant Communities

**FIGURE 1:**
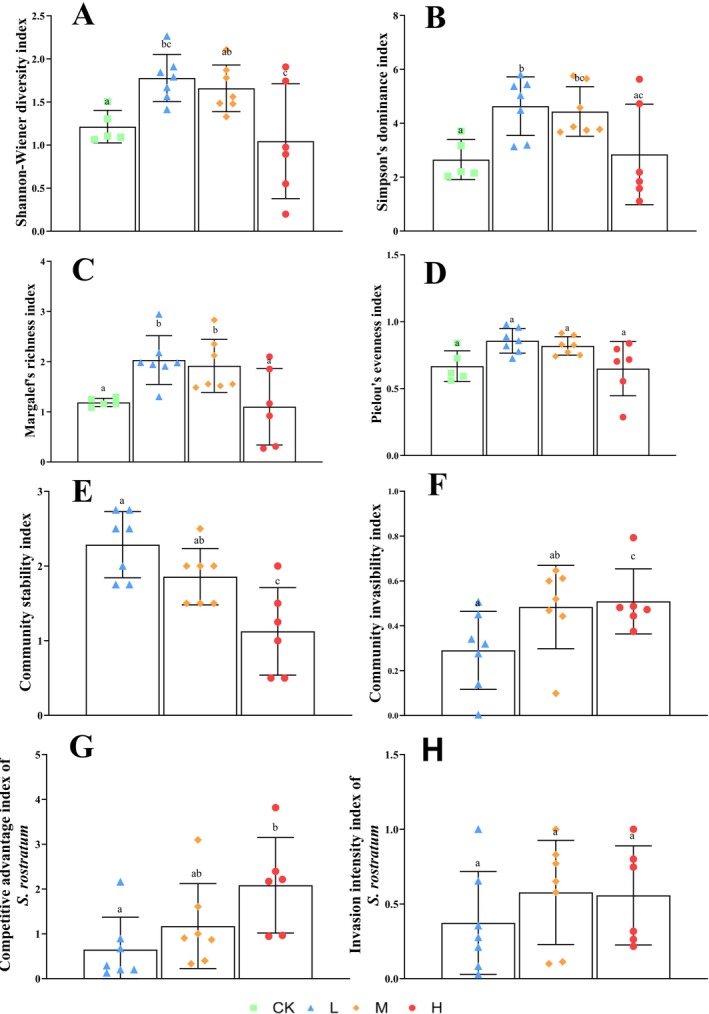
Changes in native plant community diversity indices, community stability, invasibility, and the competitive advantage index of *Solanum rostratum* along with invasion intensity under different levels of *S. rostratum* invasion. (A) Shannon‐Wiener diversity index; (B) Simpson's dominance index; (C) Pielou's evenness index; (D) Margalef's richness index; (E) community stability index; (F) community invasibility index; (G) competitive advantage index of *S. rostratum* ; (H) invasion intensity index of *S. rostratum* . Different lowercase letters indicate significant differences in species diversity indices under different invasion intensities (mean ± SD, *p* < 0.05). CK, no *S. rostratum* invasion; *H*, heavy *S. rostratum* invasion; *L*, light *S. rostratum* invasion; *M*, moderate *S. rostratum* invasion.

The invasion gradient of *S. rostratum* significantly affected diversity indices of native plant communities (*p* < 0.05). All diversity metrics exhibited unimodal response patterns along the invasion intensity gradient, characterized by initial increases followed by subsequent declines. Both Shannon's and Simpson's indices exhibited significant differences across all four invasion levels (*p* < 0.05), reaching their peak values (2.26 and 5.80, respectively) under low‐invasion conditions. Similarly, Pielou's (0.98) and Margalef's (2.94) indices were highest under low‐invasion and significantly higher in non‐invaded areas compared to heavily invaded sites (*p* < 0.05). These results suggest that low levels of invasion may temporarily enhance diversity through resource complementarity effects, while heavy invasion significantly reduces native plant community diversity (Figure 1, *p* < 0.05).

The community stability index significantly decreased with increases in the degree of invasion (*p* < 0.05). The high invasion degree (*H*) significantly reduced the community stability index by 57.8% compared with the low‐invasion degree (*L*), indicating that high‐intensity invasion severely weakened community stability. The community invasiveness and competitive advantage indices of *S. rostratum* increased significantly with the degree of invasion (*p* < 0.05). From low‐invasion (*L*) to high invasion (*H*), the community invasiveness index increased by 50.07%, and the competitive advantage index of *S. rostratum* increased by 175.36%. This indicates that a higher degree of invasion facilitates further invasions of the community and significantly enhances the competitive ability of *S. rostratum* . Although the invasion intensity index of *S. rostratum* showed an increasing trend, the difference was not statistically significant (*p* ≥ 0.05). With the progression of invasion, community invasibility increased while stability decreased (Figure 1). The nonlinear response pattern of community diversity and stability, which increases initially and then decreases with invasion intensity, and significant increase in invasibility support the diversity‐stability threshold hypothesis (Liang et al. 2025). This indicates that once the invasion degree exceeds a critical threshold, the resilience of the native community will decline sharply.

**FIGURE 2:**
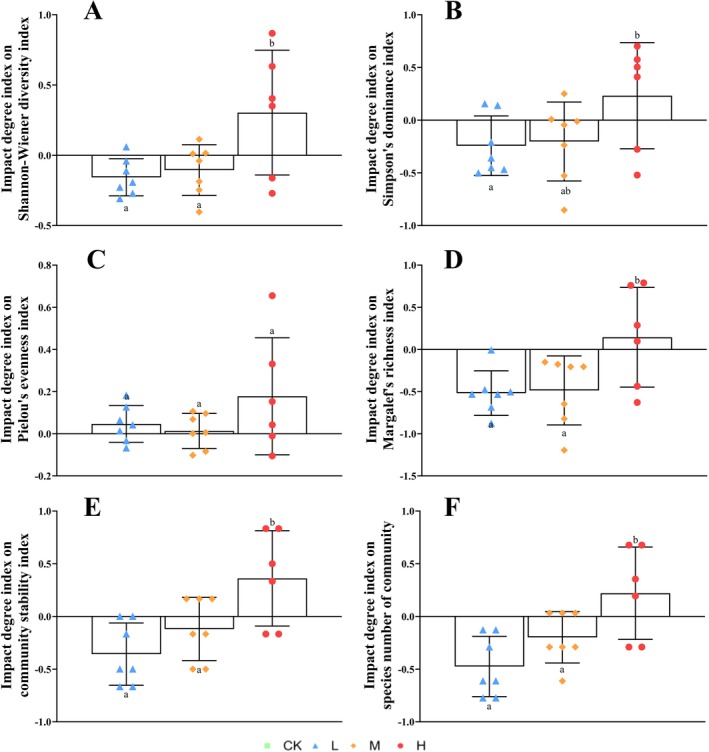
Effects *of Solanum rostratum* invasion on the impact degree indices of native plant community diversity, species number, and stability index. (A) Impact degree index on Shannon‐Wiener diversity index; (B) impact degree index on Simpson's dominance index; (C) impact degree index on Pielou's evenness index; (D) impact degree index on Margalef's richness index; (E) impact degree index on community stability index; (F) impact degree index on species number of community. Different lowercase letters indicate significant differences in impact degree indices under different invasion intensities (mean ± SD, *p* < 0.05). CK, non‐invaded control (no *S. rostratum*); *H*, heavy invasion of *S. rostratum* ; *L*, light invasion of *S. rostratum*; *M*, moderate invasion of *S. rostratum* .

The effect of *S. rostratum* invasion intensity on native plant community diversity indices showed significant differences among invasion levels (*p* < 0.05). Under high invasion (*H*), the impact degree index was positive (inhibiting local community diversity); whereas under low‐invasion (*L*) and medium invasion (*M*), both impact degree indices were negative (promoting local community diversity), indicating that low and medium invasion significantly improve local plant community diversity, with low‐invasion having the greatest effect on the Margalef richness index (impact degree index = −0.52). High invasion significantly inhibited the diversity of native plant communities (impact index > 0; Figure 2).

The invasion of *S. rostratum* significantly improved community stability and species richness (*p* < 0.05). The analysis showed that the impact index of *S. rostratum* on the community stability index was less than 0 during low and medium invasion, indicating enhanced stability. However, the impact index was > 0 during high invasion, indicating a significant decrease in stability. Moreover, the impact index of *S. rostratum* on the number of species in the community was less than 0 during low and medium invasion, indicating an increase in the number of species, whereas it decreased significantly under high invasion (Figure 2, *p* < 0.05).

### 3.3 Correlation Among Community Diversity Index, Community Stability, and Community Invasiveness

Correlation analysis was performed between diversity indices and community stability/invasibility in *S. rostratum* ‐invaded communities. All diversity indices (species richness *S*, Shannon‐Wiener index *H*′, Simpson index *D*, Pielou's evenness *J*, and functional diversity *F*) showed significant positive correlations with ICV (*p* < 0.05; correlation coefficients (*r*) = 0.96, 0.84, 0.72, 0.46, and 0.84, respectively), with *H*′, *D*, and *F* exhibiting highly significant positive correlations (*p* < 0.001). The strongest correlation was observed between *F* and *H*′ (*r* = 0.84). All diversity indices (*S*, *H*′, *D*, *J*, and *F*) displayed negative correlations with CII, CAI, and III, with *S* and *D* exhibiting significant negative correlations with CAI (*p* < 0.05, the correlation coefficients were: *r* = −0.51 and −0.53, respectively); *H*, *J*, and *F* showed highly significant negative correlations with CAI (*p* < 0.01, the correlation coefficients were: *r* = −0.63, −0.64, and −0.63, respectively); and *H* and *F* demonstrated significant negative correlations with III (*p* < 0.05, the correlation coefficients were: *r* = −0.47 and −0.47, respectively). The aforementioned studies demonstrate that community diversity exhibits a positive correlation with community stability and a negative correlation with community invasibility (Figure S1). The results showing a significant positive correlation between species diversity indices and community stability and a significant negative correlation with invasibility provide key empirical support for the diversity‐stability threshold hypothesis, thereby confirming that communities with higher diversity possess greater invasion resistance.

### 3.4 Path Analysis of Community Diversity Index, Community Stability, and Community Invasiveness

Path analysis revealed that in terms of the contributions to community stability (Table 4), the species diversity indices ranked as follows: Species richness (*S*) > Shannon‐Wiener index (*H*′) > Pielou's evenness index (*J*) > Margalef richness index (*F*) > Simpson's dominance index (*D*). Species richness (*S*) had the strongest direct contribution (*β* = 0.736). *S* also exerted a substantial indirect contribution via *H*′ (*β*′ = 00.661). In terms of community invasibility, the ranking of contributions was as follows: Species richness (*S*) > Shannon‐Wiener index (*H*′) > Margalef richness index (*F*) > Simpson's index (*D*) > Pielou's index (*J*). Species richness (*S*) again showed the greatest direct effect (*β* = 0.701). S had a notable indirect effect through F (*β*′ = 0.633, Table 4). Path analysis further indicated that species richness had the most direct contribution to both community stability and invasibility, reinforcing the view that species diversity acts as a core factor driving invasion outcomes and reaffirming the key prediction of the diversity‐stability threshold hypothesis.

**TABLE 4:** Contributions of plant community diversity to community stability and invasibility under *Solanum rostratum* invasion.

Community stability index							Community invasiveness index					
	*β*	*β*′					*β*	*β*′				
		*S*	*H*′	*D*	*J*	*F*		*S*	*H*′	*D*	*J*	*F*
*S*	0.736	—	0.661	−0.168	0.359	−0.084	0.701	—	−0.632	0.574	−0.362	−0.633
*H*	0.749	0.650	—	−0.200	0.518	−0.090	−0.573	0.517	—	0.540	−0.454	−0.541
*D*	−0.213	0.579	0.703	—	0.508	−0.087	0.200	0.164	−0.188	—	−0.153	−0.180
*J*	−0.339	0.403	0.592	−0.165		−0.063	−0.154	0.080	−0.120	0.118	—	−0.102
*F*	−0.096	0.646	0.704	−0.193	0.430	—	−0.237	0.214	−0.224	0.214	−0.156	—

Abbreviations: *β*, direct path coefficient; *β*′, indirect path coefficient; *D*, Simpson's dominance index; *F*, Margalef's richness index; *H*′, Shannon‐Wiener diversity index; *J*, Pielou's evenness index; *S*, number of plant species in the community.

### 3.5 Impact of *S. rostratum* on the Functional Diversity of Native Plant Communities

*S. rostratum* invasion significantly altered native plant functional traits (*p* < 0.05), with heavy invasion increasing leaf length and plant height and decreasing the aboveground biomass relative to those of the non‐invaded areas. Aboveground biomass of native plants. However, other traits such as leaf thickness and leaf width did not significantly differ with increasing invasion degree (Table S1). *S. rostratum* exhibited significant trait plasticity across invasion intensities (*p* < 0.05), with heavy invasion enhancing leaf dimensions (width, thickness, and area), plant height, and the aboveground biomass compared with low levels of invasion (Table S2).

**FIGURE 3:**
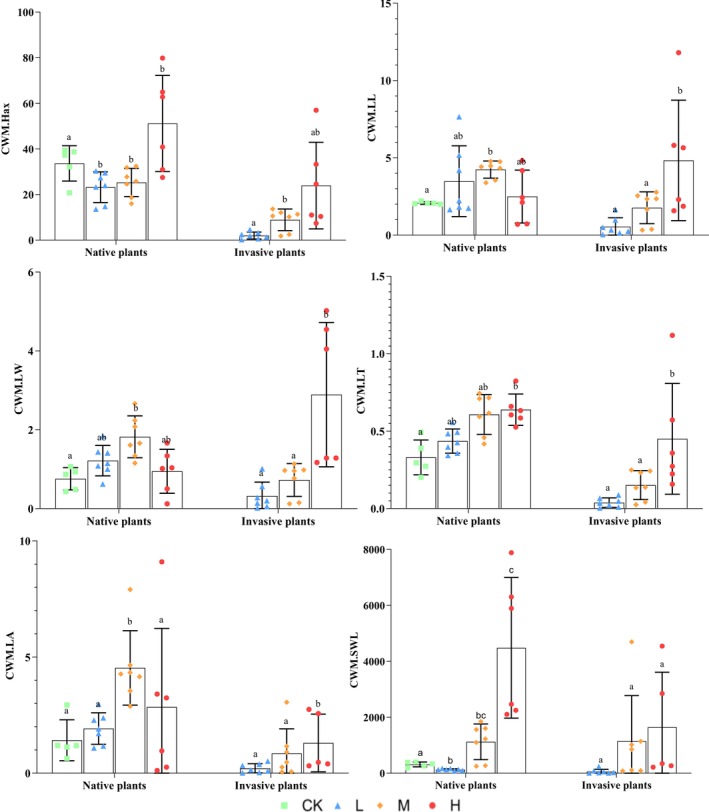
Weighted mean trait indices of native versus invasive plants in plant communities under different *Solanum rostratum* invasion intensities. CK, no invasion of *S. rostratum* ; CW.PHax, community‐weighted mean trait value of maximum plant height; CWM.LA, community‐weighted mean trait value of leaf area; CWM.LL, community‐weighted mean trait value of leaf length; CWM.LT, community‐weighted mean trait value of leaf thickness; CWMLW, community‐weighted mean trait value of leaf width; CWM.SWL, community‐weighted mean trait value of specific leaf area; *H*, severe invasion of *S. rostratum* ; *L*, light invasion of *S. rostratum* ; *M*, moderate invasion of *S. rostratum* . Different lowercase letters indicate statistically significant differences (*p* < 0.05).

The invasion not only significantly enhanced the trait expression of the invasive plant itself but also exerted complex effects on the functional trait composition of the native plant community (*p* < 0.05 Figure 3); with increasing invasion intensity of *S. rostratum* , the community‐weighted mean trait indices of native and invasive plant communities exhibited significant divergence. After the invasion of *S. rostratum* , the CWM.Hmax showed an initial decline followed by an increase, while CWM.LL, CWM.LW, and CWM.LA displayed an initial rise followed by a decrease. In contrast, CWM.LT and CWM.SWL demonstrated a significant increasing trend. Meanwhile, all community‐weighted mean values of the invasive plants exhibited a significant upward trend.

The invasion of *S. rostratum* significantly altered the community‐weighted mean trait values (*p* < 0.05; Table S3). Specifically, the (1) leaf morphological traits (width, thickness, and area) increased with invasion intensity; (2) plant height initially decreased then increased with invasion intensity; and (3) aboveground biomass of *S. rostratum* increased with invasion intensity while that of native plants decreased.

**FIGURE 4:**
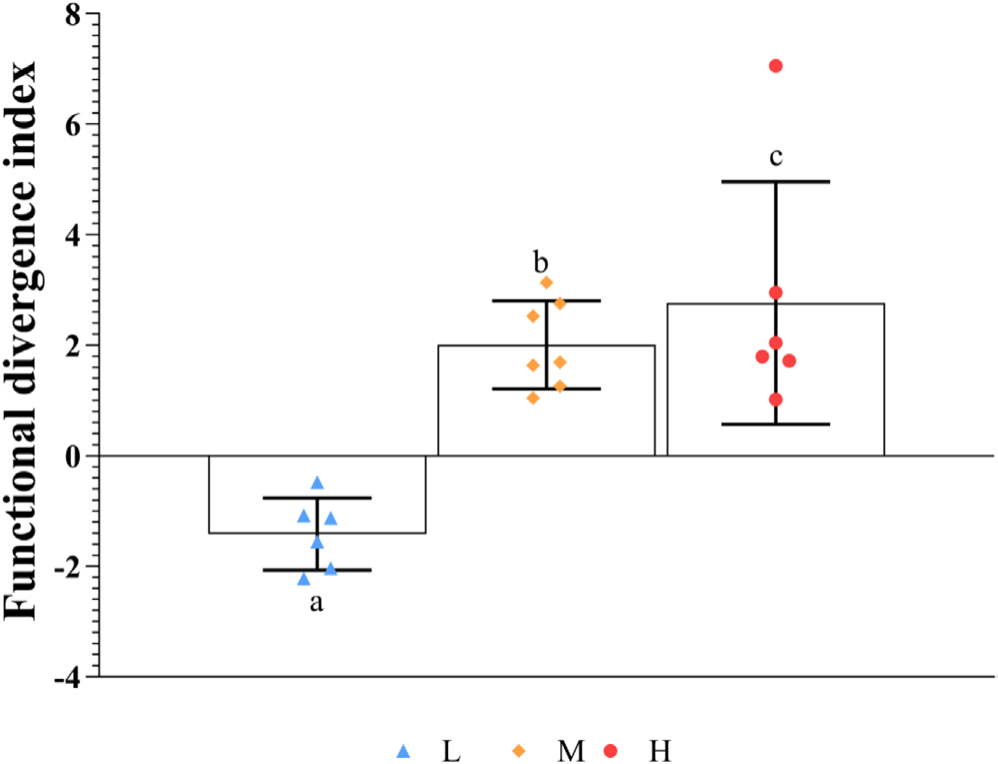
Functional divergence indices of traits between *Solanum rostratum* and native plants. *H*, severe invasion of *S. rostratum*; *L*, light invasion of *S. rostratum* ; *M*, moderate invasion of *S. rostratum* (*p* < 0.05).

Analysis of the functional difference index showed that the invasion degree of *S. rostratum* significantly affected its functional relationship with local plants (*p* < 0.05). Specifically, in mildly invaded plots, the functional divergence index between *S. rostratum* and native plants was negative but shifted to positive in moderately and heavily invaded plots (Figure 4). Compared with the low‐invasion plot, the average functional difference index significantly increased in the medium‐ and high‐invasion plots (Figure 4, *p* < 0.05). The functional difference index shifted from negative values under low invasion to positive values under moderate‐to‐high invasion. This dynamic change strongly supports the functional trait replacement hypothesis (Bjorkman et al. 2018), indicating that *S. rostratum* alters the functional structure of the community through competitive exclusion and trait replacement.

**FIGURE 5:**
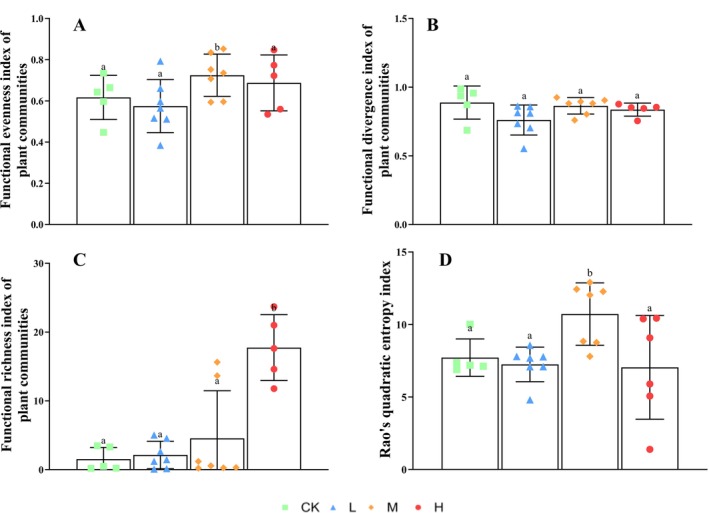
Functional evenness (FEve), functional divergence (FDiv), functional richness (FRic), and Rao's quadratic entropy (RaoQ) of plant communities under different invasion intensities of *Solanum rostratum* . (A) Functional evenness index of plant communities; (B) functional divergence index of plant communities; C, functional richness index of plant communities; (D) Rao's quadratic entropy index.

**FIGURE 6:**
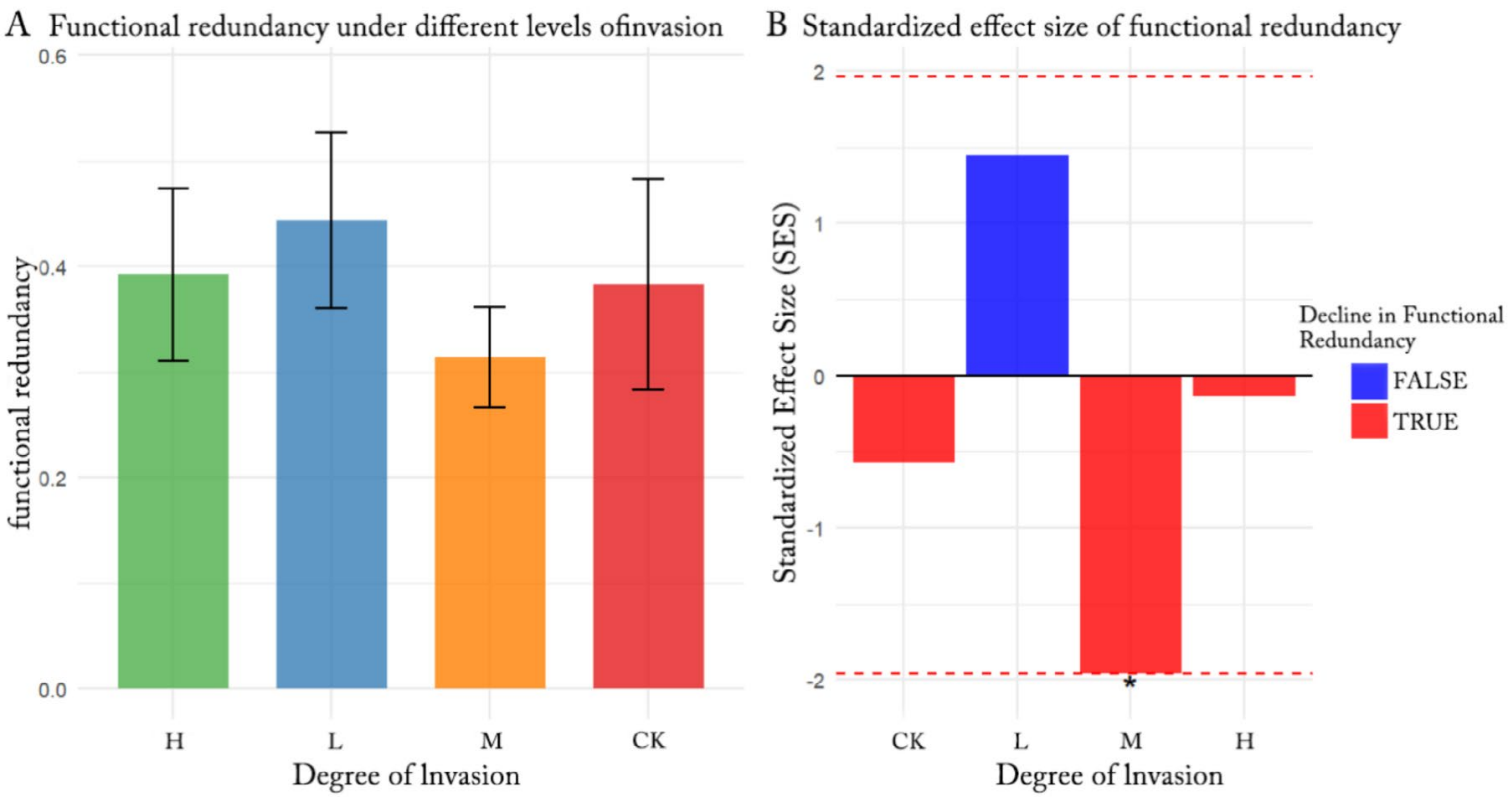
Analysis of the functional redundancy null model for *Solanum rostratum* under different invasion degrees. Changes in plant community functional redundancy under different levels of invasion. (A) Variation in functional redundancy across degrees of invasion (CK, no invasion; *H*, high invasion; *L*, low invasion; *M*, moderate invasion). (B) Standardized effect size (SES) of functional redundancy, where dashed lines indicate the significance threshold of ±1.96 (i.e., SES values beyond this range indicate statistically significant changes in functional redundancy). The figure illustrates a significant decline in functional redundancy with increasing invasion intensity.

Specifically, the FEve (Functional evenness index of plant communities) index significantly increased in the medium‐invasion plot of *S. rostratum* but did not significantly differ in the low‐ and high‐invasion plots. Moreover, the FDiv (Functional divergence index of plant communities) index did not significantly differ in the low‐, medium‐, and high‐invasion plots. The FRic (Functional richness index of plant communities) index of the high‐invasion plot of *S. rostratum* significantly increased, while it did not significantly differ in the low‐ and medium‐invasion plots. RaoQ (Rao's quadratic entropy index) significantly increased in the medium‐invasion plot but did not significantly differ in the low‐ and high‐invasion plots (Figure 5). Therefore, it can be concluded that medium invasion promotes functional balance allocation and differential evolution, while high invasion changes the functional structure through niche reconstruction and competitive screening. The significant increase in the functional diversity index under high invasion levels and phased changes in functional evenness and Rao's Q index at moderate invasion levels align with the predictions of the functional redundancy loss hypothesis (Hatton et al. 2024). This suggests that as invasion intensifies, the loss of native species leads to a collapse in functional redundancy (Figure 6), resulting in diminished community resistance to disturbance.

### 3.6 Correlation Analysis Between Diversity Index and Functional Diversity Index of Native Plant Communities Invaded by *S. rostratum*

The interplay between community diversity metrics and functional diversity in native vegetation under *S. rostratum* invasion indicates that the Shannon diversity (*H*′) and functional diversity (*F*) indices were significantly negatively correlated with the CWM of the functional diversity index (*p* < 0.001), whereas the Simpson diversity (*D*) and Pielou's evenness (*J*) indices were significantly negatively correlated with the CWM of the functional diversity index (*p* < 0.01), with the highest negative correlation coefficient (0.72) between the *H*′ of community diversity and CWM. The community diversity indices *H*′ and *J* were highly significantly positively correlated with the functional diversity index FDQ (*p* < 0.01), while the community diversity indices *D* and *F* values were positively correlated with the FDQ (*p* < 0.05); however, CWM was negatively correlated with FDQ. Overall, a negative correlation pattern was observed between the community species diversity and functional diversity (CWM) indices (Figure S2).

### 3.7 The Impact of *S. rostratum* Invasion at Different Intensities on the Functional Redundancy of Native Plants

This study employed null model analysis to reveal that the impact of *S. rostratum* invasion on the functional redundancy of local plant communities exhibits pronounced nonlinear dynamics. The results indicate that moderate invasion represents a critical stage leading to significant loss of functional redundancy in local plant communities (Figure 6).

Specifically, during the uninvaded (CK) and low‐invasion (*L*) stages, the functional redundancy of local plant communities showed no significant deviation from random expectations (SES = −0.571, *p* = 0.264; SES = 1.448, *p* = 0.923). However, at the moderate‐invasion (*M*) stage, a marked loss of functional redundancy was observed (SES = −1.96, *p* = 0.027). This significant negative SES value indicates that the observed reduction in functional redundancy far exceeds expectations from random processes. This may be attributed to the invasive species reaching a critical population size at this stage, systematically outcompeting or displacing native species within key functional groups. Such displacement reduces species richness within these groups, thereby weakening the ecosystem's buffering capacity against disturbances. During the high‐invasion (*H*) stage, functional redundancy remained lower than levels observed under low invasion but did not differ significantly from random expectations (SES = −0.141, *p* = 0.463). These findings demonstrate that *S. rostratum* invasion does not uniformly diminish functional redundancy across all stages but exerts its most pronounced negative effects specifically during the moderate‐invasion phase.

## 4 Discussion

This study reveals the complex mechanisms by which *S. rostratum* invasion affects plant communities. As the invasion intensity increases, plant community diversity and stability decrease. The invasibility of plant communities showed a negative correlation with diversity and stability, supporting the “diversity resistance hypothesis”—that species diversity is negatively correlated with invasibility. However, the effects varied depending on invasion intensity: low‐invasion plots exhibited increased species diversity indices compared to control plots. In contrast, varying invasion intensities differentially impacted native plant community diversity. Compared to non‐invaded sites, low‐intensity invasion significantly increased species diversity indices (*p* < 0.05). However, as invasion intensity and *S. rostratum's* competitive advantage intensified, native community diversity indices exhibited a significant declining trend. This finding aligns with multiple studies by Vilà et al. (2011). Liu et al. analyzed the population characteristics of *A. artemisiifolia* under different invasion pressures and found that increasing invasion intensity first enhanced but later reduced plant species diversity (Liu et al. 2021). Similarly, Lu et al. observed in Shenzhen that low‐invasion plant plots had higher Simpson dominance index, Shannon‐Wiener diversity index, Margalef richness index, and Pielou evenness index compared to heavily invaded and non‐invaded plots (Lu et al. 2023). The above results may be explained as follows: during the early stages of invasion, invasive plants did not significantly displace native species in the community and thus exhibited species diversity similar to that of non‐invasive communities (Zhang et al. 2024). However, in the later stages of invasion, the invasive plants strengthened their competitive advantage through differentiation strategies. The study found that low‐level invasion by *S. rostratum* can enhance the diversity of native plant communities.

Community invasibility is a key determinant for successful alien plant colonization. The study demonstrates that invasibility shows a significant positive correlation with *S. rostratum* coverage (*r* = 0.68, *p* < 0.01) and negative correlations with both community diversity and stability. With an increase in invasive plant coverage, the invasiveness of the community showed an upward trend, while the diversity and stability showed a downward trend. This result supports the “diversity impedance hypothesis” and Livingstone et al. (2020) reached the same conclusion in their study on the reduction of species diversity in native plant communities by the invasion of *Galinsoga parviflora* . Wang et al. (2015) also found that as the invasion of *Ageratina adenophora* increased, the diversity of local plant species decreased. The diversity impedance hypothesis suggests that compared with ecosystems with lower biodiversity, ecosystems with higher biodiversity have higher stability and less invasiveness; thus, it is difficult for invasive plants to successfully invade ecosystems with higher biodiversity. Invasive plants exhibit strong adaptability in low‐resource environments and occupy local plant ecological niches through rapid colonization and expansion, thereby competing for key resources such as light, water, and nutrients, ultimately disrupting the ecosystem balance, and this may cause the extinction of other plants (Park et al. 2022). With the increasing invasion intensity of *S. rostratum* , its impacts on native plant community diversity and stability progressively intensify. This pattern aligns with findings on *Xanthium italicum* invasion, where greater invasion degrees similarly amplified ecological impacts on plant communities (Xiao et al. 2023). The escalating effects stem from three synergistic mechanisms: enhanced competitive superiority and invasion vigor at higher invasion intensities, increased resource acquisition and niche occupation, and elevated allelochemical production. Together, these processes disrupt native community structure and diminish biodiversity, exacerbating ecological consequences. A negative correlation exists between plant community diversity/stability and community invasibility. Consequently, reduced diversity and stability enhance community invasibility, intensifying invasion pressure, ultimately establishing a vicious cycle that facilitates alien species invasion (Xiao et al. 2023).

As a core indicator for characterizing community biodiversity, plant species richness exhibits fundamental connections with native plant community diversity and constitutes its basic structural attribute (Mata et al. 2013). Community stability and invasiveness are closely related to local plant community diversity (Henriques and Hay 2002; Levine and D'Antonio 1999; Xiao et al. 2023). Our experimental results demonstrate that both species richness and the Shannon‐Wiener index contribute significantly more to community stability than other diversity metrics, consistent with the findings of Wang et al. (2021). This indicates that communities with higher species richness and Shannon‐Wiener indices exhibit greater stability and enhanced resistance to plant invasions. Furthermore, these two indices also show superior explanatory power for community invasibility, compared with other diversity parameters, a conclusion that corroborates research by Yang et al. (2019). This indicates that as the degree of *S. rostratum* invasion increases, it becomes the dominant species in the community, increasing its competitive advantage and invasion intensity. The species richness and Shannon‐Wiener index play pivotal roles in this process. Consequently, species richness and the Shannon‐Wiener index significantly influence community stability and invasibility.

Plant functional traits may exhibit significant variations under different environmental conditions, thereby reflecting their adaptive strategies to environmental changes (Cooper 2018; Gallagher et al. 2015; Yang et al. 2019). In different growing environments, *Solanum carolinense* exhibits different growth strategies through phenotypic plasticity. The results of this study demonstrate significant differences in functional traits between *S. rostratum* and co‐occurring native plants across varying invasion intensities. Specifically, leaf width, thickness, area, and plant height of *S. rostratum* showed significant increases with escalating *S. rostratum* invasion intensity. The leaf length, leaf thickness, and leaf area of *S. rostratum* consistently exceed those of native plants across all invasion stages. This may be because, as the degree of *S. rostratum* invasion increases, *S. rostratum* competes to exclude local species, using its competitive advantages (morpho‐functional plant traits, including height and leaf area parameters) to obtain more resources for better diffusion and colonization. Thus, it forms a single dominant population that occupies more ecological niches and continuously reduces the dominant position of local plants coexisting with it at the invasion site. The relevant research results indicate that the functional traits of plants vary with different degrees of plant invasion, which is associated with different environmental factors (Wang et al. 2021; Zenni et al. 2020). Wang et al. (2021) found that as the invasion of *Cenchrus pauciflorus* increased, local plants also experienced an accelerated decrease in species diversity, which is consistent with the above conclusion. The underlying mechanism may lie in the critical role of functional height in resource competition among plants (Rolhauser et al. 2019), where tall‐statured invasive species suppress native plant growth through shading.

Light is an indispensable resource for plant growth and represents the most critical environmental factor influencing plant development. As *S. rostratum* invasions increase, both the invasive plant and co‐occurring native species exhibit an initial decrease followed by a subsequent increase in plant height. Notably, *S. rostratum* experiences significant height augmentation with different invasion levels, and its plant height consistently exceeds that of native plants across all invasion gradients, a morphological adaptation that enhances light acquisition competitiveness. This adaptive strategy may ultimately drive functional trait divergence between the invader and native flora (Rolhauser et al. 2019). Research has indicated that as the invasion level increases, invasive plants can expand their populations through rapid reproduction strategies to enhance their competitive advantages over limited resources. In addition, the nutrient allocation strategy of *S. rostratum* can be adjusted according to environmental changes.

The study found that the increasing invasion intensity of *S. rostratum* increased community‐weighted mean indices of invasive plants and resulted in significant increases in leaf dimensions (length and width) and plant height. The competitive ability of species and the rate of resource use can be characterized by the size of the plant leaves (leaf length and width) (Petchey and Gaston 2006; Westerband et al. 2025). Therefore, changes in the environment (an increase in the level of invasion) enable invasive plants to enhance their competitive advantage for environmental resources, resulting in changes to the functional traits, such as leaf width, plant height, and aboveground total biomass.

The results clearly demonstrate that *S. rostratum* exhibits a significant increase in aboveground biomass under moderate and heavy invasion levels compared to light invasion (Table S2). This pattern of escalating biomass accumulation with invasion intensity underscores the invader's enhanced capacity for resource acquisition and growing competitive dominance within the community. In stark contrast, the total aboveground biomass of native plant communities displayed a declining trend under high invasion pressure (Table S1), signaling a process of progressive competitive exclusion as *S. rostratum* monopolizes resources. These complementary biomass dynamics between the invader and natives reinforce the functional trait replacement hypothesis, illustrating how *S. rostratum* effectively outcompetes and displaces native species through its superior growth and resource preemption strategies (which is also reflected in its greater plant height and leaf area, Table S2). The biomass data thus offer a direct quantitative link between the invader's functional advantages, the restructuring of soil nutrient regimes (Table 3), and the eventual decline in native community diversity and stability. This empirical evidence solidifies our understanding of the central resource competition mechanism underlying the successful invasion of *S. rostratum* . Research on *S. carolinense* also supports this point (Wise and Abrahamson 2007).

Plant functional diversity, as an important component of biodiversity, is reflected in the easily measurable morphological, chemical, physiological, and phenological properties exhibited by plants through long‐term adaptations to their surrounding environment. Thus, diversity can reflect the structure and function of ecosystems (Guo et al. 2024; Loreau et al. 2001). The results showed that it significantly increased under high invasion intensity relative to low invasion intensity, and the FEve and RaoQ indices significantly increased under medium invasion levels and then returned to baseline; the FDiv index showed no significant change, while the FRic index increased with the degree of invasion. The invasion of *S. rostratum* can have a significant effect on the functional diversity of plant communities; however, this effect is not a simple linear process. Instead, at medium invasion, it reshapes the functional structure of the community in stages by increasing plant height and leaf thickness and by other factors. During the transition from a low‐invasion to high‐invasion plot of *S. rostratum* , the functional similarity between species in the community increased, leading to increasingly fierce competition and challenges in terms of species coexistence, resulting in exclusion and ecological niche differentiation (Gross et al. 2013; Zhang et al. 2015). This result supports the selection effect hypothesis, which suggests that due to the differentiation of biological niches, ecosystems with high biodiversity are better able to use environmental resources than those with low biodiversity. With an increase in biodiversity, the probability of dominant species with excellent functional traits appearing in the ecosystem also increases, thus increasing the functional diversity of the ecosystem. In the low‐invasion plot, *S. rostratum* and its coexisting native plants attain functional equivalence in terms of resource utilization. During medium to high invasion stages, *S. rostratum* exhibited significantly superior functional traits compared with native plants, with evident functional divergence between them. Consequently, *S. rostratum* enhanced the functional diversity of native plant communities during this phase, which not only improved the efficiency of resource utilization within the community (Gazol and Camarero 2016; McGill et al. 2006) but also facilitated more stable population expansion and reproduction of the invader.

The observed negative correlation between species diversity and functional diversity contrasts with classical ecological expectations and reveals distinctive community restructuring under *S. rostratum* invasion. The negative correlation between species diversity and functional diversity (CWM) indicates that species loss and functional trait reassembly are not synchronous in the context of invasion. This complex relationship suggests that invasion by *S. rostratum* not only reduces species richness but, more critically, drives functional filtering and reorganization of traits. These findings provide deeper evidence supporting the functional trait replacement hypothesis. Moreover, the findings reveal the complex impact mechanism of *S. rostratum* invasion on plant communities. Different degrees of *S. rostratum* invasion significantly affect plant community diversity and stability, plant community functional diversity, and soil nutrient content. The invasion of *S. rostratum* reconstructs the soil nutrient pattern, which shows phase‐specific disturbance characteristics. As the degree of invasion intensifies, the soil carbon (C) and nitrogen (N) content continue to significantly decrease, indicating that invasive plants continue to deplete the soil carbon and nitrogen pool through efficient absorption and other life activities. During mild invasion, nitrogen form transformation presents a critical turning point, with nitrate nitrogen content decreasing to 0.0048 g/kg, representing an 83% reduction, and ammonium nitrogen increasing to 0.062, representing a 63% increase. This may be due to the inhibition of nitrification by root exudates of *S. rostratum* , thereby leading to a shift in nitrogen cycling from nitrification to ammonium accumulation mode. During severe invasion (*H*), the phosphorus (P) content increased to 0.62, which was significantly higher than that of the mild invasion stage of 0.55. This may be related to the significant increase in the number of *S. rostratum* due to the extinction of local plants.

This pattern reflects a disturbance‐driven shift in diversity‐function relationships, thereby signaling early ecosystem destabilization. Two mechanisms explain this phenomenon: First, functional redundancy collapse and trait dominance. Under light invasion, high diversity and functional redundancy are initially maintained. Under intense invasion, competitive exclusion reduces redundancy while *S. rostratum* 's extreme traits (e.g., height, leaf area) expand functional space boundaries, thereby increasing metrics like FRic despite species loss (Figures 5 and 6; Tables S1 and S2). This represents a vulnerable invader‐driven “false prosperity.” Second, functional divergence and community reassembly. The FDj shift from negative to positive with invasion intensity (Figure 4) indicates strategy divergence and niche differentiation among survivors, thereby forcibly altering the functional space and diversity‐function linkages. These findings align with those of Xiao et al. (2023) on invasion‐induced functional reorganization.

In addition, research has shown that the roots of *S. rostratum* harbor a rich and diverse fungal community and the secondary metabolites secreted by the roots can influence the surrounding environment through allelopathy. The findings of this study indicate that as the degree of *S. rostratum* invasion intensifies, the soil nutrient content undergoes corresponding changes. That *S. rostratum* exhibits high plasticity in nitrogen uptake forms. Compared to coexisting native plants, its growth advantage may stem from its stronger nitrogen absorption capacity and higher nitrogen utilization efficiency. This study further reveals that during the early stage of invasion, *S. rostratum* temporarily enhances the availability of soil nutrients by promoting the transformation of nitrogen forms (during light invasion, the ammonium nitrogen content significantly increases while the nitrate nitrogen content significantly decreases). At this time, the diversity index of the native plant community is significantly higher than that in the non‐invaded stage. However, during moderate to severe invasion periods, the continuous depletion of carbon and nitrogen pools leads to a decline in soil carrying capacity. By the severe invasion stage, plant community diversity collapses and the system stability drops sharply. The gradual degradation of the soil nutrient structure, particularly the continuous loss of carbon and nitrogen as well as the dynamic reconfiguration of phosphorus, provides empirical evidence for elucidating the dual mechanism of “resource plundering‐niche reconstruction” that drives ecosystem degradation during the invasion process of *S. rostratum* .

The findings of this study provide strong empirical support for validating the three core theoretical hypotheses driving plant invasion.

Support for the diversity‐stability threshold hypothesis: low‐level invasion significantly enhances the species diversity and stability of the native plant community, potentially through resource complementarity (Figure 1, *p* < 0.05). However, once invasion intensity exceeds a critical threshold (moderate to high invasion), both metrics decline nonlinearly and significantly (Figures 1 and 2). This characteristic pattern of “facilitation‐to‐inhibition” directly validates this hypothesis (Liang et al. 2025), highlighting the ecological management importance of intervention before the threshold is breached.

Support for the functional trait replacement hypothesis: the data strongly support this hypothesis. The CWM of key functional traits (e.g., plant height and leaf area) for the invasive species *S. rostratum* was consistently significantly higher than that of native species (Figure 4, Tables S2 and S3). More importantly, the functional divergence index between the invader and native species shifted from negative to positive with increasing invasion intensity (Figure 5). This indicates a shift in invasion strategy from initial functional convergence to later functional divergence, thereby achieving niche occupation and trait replacement through superior resource‐acquisition traits (Bjorkman et al. 2018).

Support for the functional redundancy loss hypothesis: the decoupled response patterns of species diversity and functional diversity provide key evidence for this hypothesis. This suggests the collapse of functional redundancy, where community function becomes dominated by the traits of the invasive dominant species, thereby increasing ecosystem risk (Hatton et al. 2024).

This study demonstrates a negative correlation between species diversity and functional diversity in native plant communities. Previous studies have identified four principal conclusions regarding species‐function relationships in plant communities: (1) positive species‐functional diversity correlation (Liu et al. 2013; Mason et al. 2005); (2) negative species‐functional diversity correlation (Sasaki et al. 2009), which is consistent with our findings; (3) nonsignificant species‐functional diversity correlation; and (4) an S‐shaped species‐functional diversity correlation (Bu et al. 2014). These discrepancies may be due to differences in the structure, number of species, size, and functional types of the studied plant communities, indicating that the relationship between species diversity and functional diversity in plant communities is complex (Bu et al. 2014) and any factor within a plant community can impact the relationship.

This study systematically reveals the stage‐specific impacts of *S. rostratum* invasion on plant community diversity, functional traits, and soil nutrients using a space‐for‐time substitution approach. Based on three core hypotheses (diversity‐stability threshold, functional trait replacement, and loss of functional redundancy), potential ecological mechanisms such as resource complementarity, soil nutrient restructuring, and competition strategy shifts are proposed. However, due to limitations in the research design (e.g., inferring temporal dynamics from spatial gradients, single‐point sampling, etc.), the mechanisms proposed in this paper largely represent reasonable inferences drawn from correlation analyses rather than causal relationships verified through controlled experiments. For example, promotion of diversity by the resource complementarity mechanism during the early invasion stage still requires direct validation through resource addition or exclusion experiments (such as nitrogen/phosphorus fertilization or shading treatments); the causal relationship between soil nutrient restructuring and plant functional trait changes needs further clarification via soil transplantation or root‐box experiments; and the driving mechanisms behind the shift in competition strategies (from facilitation to suppression) need to be quantified by combining greenhouse competition experiments or in situ removal experiments to measure interspecific competition intensity. The study also found that drought and grazing directly and indirectly facilitate the invasion of *S. rostratum* (Shi et al. 2025). Furthermore, since the findings of this study are derived from a single geographical region (Urumqi, Xinjiang), their generalizability needs to be tested in more diverse ecosystems. Future research should prioritize long‐term fixed‐position monitoring or invasion process simulation experiments to directly capture dynamic mechanisms, multi‐regional comparative studies to test the universality of the conclusions, and the integration of multi‐dimensional data (functional traits, soil microbes, plant physiology, etc.) with molecular ecology approaches to deeply unravel the driving mechanisms of invasion. These efforts will help advance the field from “pattern recognition” to “mechanism verification,” thus providing a more solid empirical foundation for the theory of invasion ecology.

## 5 Conclusion

This study has revealed that in low‐invasion plots, *S. rostratum* temporarily enhances plant community diversity through resource‐use complementarity or niche filling, consequently improving community stability while reducing invasibility. However, with increasing invasion intensity, competitive exclusion becomes predominant, which reduces native species, community diversity, and stability and increases invasibility. This model supports the “diversity‐stability threshold hypothesis,” which posits a critical point at which species diversity promotes stability, and when the threshold is exceeded, the negative impact of invasive species intensifies. With low levels of *S. rostratum* invasion, the mean functional divergence index between the invader and co‐occurring native plants showed negative values, indicating convergent functional trait evolution. In contrast, medium‐to‐high invasion intensities promoted functional divergence between *S. rostratum* and native species, supporting the functional trait replacement hypothesis. Furthermore, a negative correlation was observed between species diversity and functional diversity in plant communities. Notably, while the functional diversity of invaded communities increased with escalating *S. rostratum* invasion intensity, this trend contrasted with the decrease in species diversity pattern in native plant communities. This indicates that the dominant species in the community contribute significantly more to plant functional diversity than other species, thus providing empirical support for the functional redundancy loss hypothesis.

The invasion of *S. rostratum* has immeasurable effects on the soil nutrient content, species and functional diversity of fragile ecosystems and native plant communities in Xinjiang. Prevention and control of *S. rostratum* should be implemented according to the degree of invasion. For example, low‐invasion areas should promote local plant diversity, supplement local plant species to consolidate community stability, and quickly remove invasive species through manual extraction or chemical control to avoid further expansion of *S. rostratum* . In addition, medium‐ and high‐invasive areas should adopt strong intervention measures to reduce the density of *S. rostratum* . Then local species with rich functional groups should be introduced to accelerate niche filling and block their competitive exclusion effects. However, given the limitations of the space‐for‐time substitution design and single‐site sampling in this study, the universality of the above conclusions and the certainty of causal relationships still require further verification through long‐term monitoring and multi‐site comparative research.

Future research should integrate multi‐regional comparative studies and long‐term in situ monitoring to systematically elucidate the spatial heterogeneity and temporal dynamics of *S. rostratum* invasion effects. Concurrently, multi‐dimensional functional trait analyses (including physiological, morphological, and chemical characteristics) of *S. rostratum* should be combined with molecular ecological techniques to investigate the underlying mechanisms driving changes in native plant species diversity. Building on these findings, a differentiated ecological management strategy framework should be developed to address varying invasion stages and ecosystem vulnerabilities, thereby enhancing targeted control and restoration practices.

## Author Contributions

**Lijun Hu:** conceptualization (equal), data curation (equal), formal analysis (equal), investigation (equal), methodology (equal), writing – original draft (equal). **Lamei Jiang:** conceptualization (equal), data curation (equal), methodology (equal), supervision (equal), writing – original draft (equal). **Juan Qiu:** conceptualization (equal), data curation (equal), funding acquisition (equal), methodology (equal), project administration (equal), resources (equal), supervision (equal). **Amanula Yimingniyazi:** conceptualization (equal), funding acquisition (equal), investigation (equal), methodology (equal), project administration (equal), resources (equal), supervision (equal), writing – review and editing (equal).

## Funding

This work was supported by the Major Science and Technology Public Relations Project Fund of the Science and Technology Department of Xinjiang Uygur Autonomous Region (2023A02006).

## Conflicts of Interest

The authors declare no conflicts of interest.

## Supporting information


**Figures S1–S2:** ece372910‐sup‐0001‐Figures.docx.

**Tables S1–S3:** ece372910‐sup‐0002‐Tables.docx.

## Data Availability

The data supporting the findings of this study are publicly available in Mendeley Data under reference number: LiJun Hu (2025), “2024 Survey Data on the Invasive Plant *Solanum rostratum* ,” Mendeley Data, V1, https://doi.org/10.17632/26xf2vm3zd.1. Related Links: https://data.mendeley.com/datasets/26xf2vm3zd/1.
