# Supplementary material for: Ecological Effects of Solanum rostratum Invasion on the Diversity and Functional Traits of Native Plant Communities
Source: Ecol Evol. 2026 Jan 11;16(1):e72910. doi: 10.1002/ece3.72910 (PMC12793780; doi:10.1002/ece3.72910)
Supplement: Supplementary file 1 — Figures S1–S2: ece372910‐sup‐0001‐Figures.docx. [file ECE3-16-e72910-s001.docx]

**
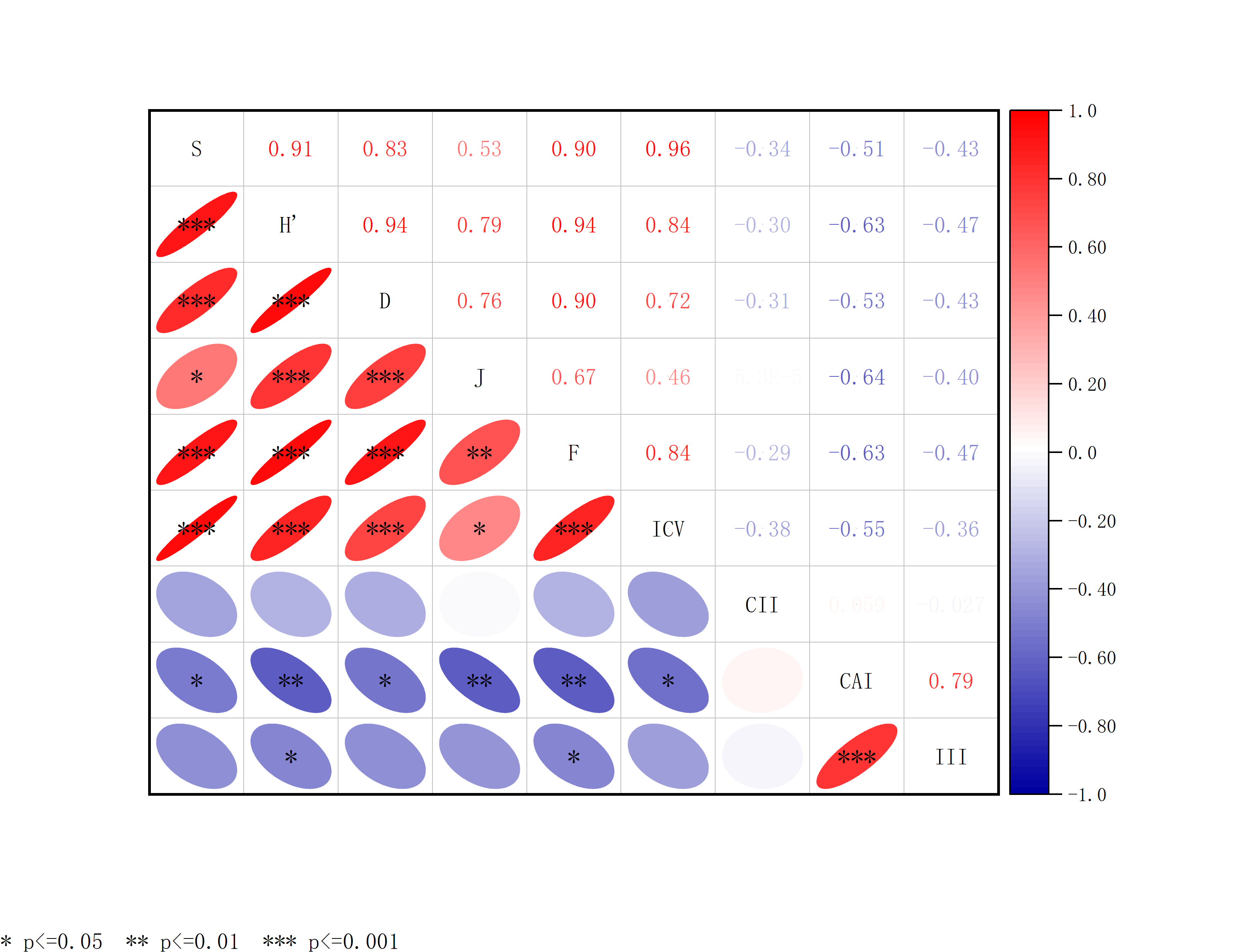
Figure 1 The correlation between the local plant community diversity index and community stability and invasibility.**

* p＜=0.05 **p＜=0.01 ***p＜=0.001

Note: S: Number of plant species; H': Shannon-Wiener diversity index; D: Simpson's dominance index; J: Pielou's evenness index; F: Margalef's richness index; ICV: Community stability index; CII: Community invasibility index; CAI: Competitive advantage index of *S. rostratum*; III: Invasion intensity index of *S. rostratum*.

**
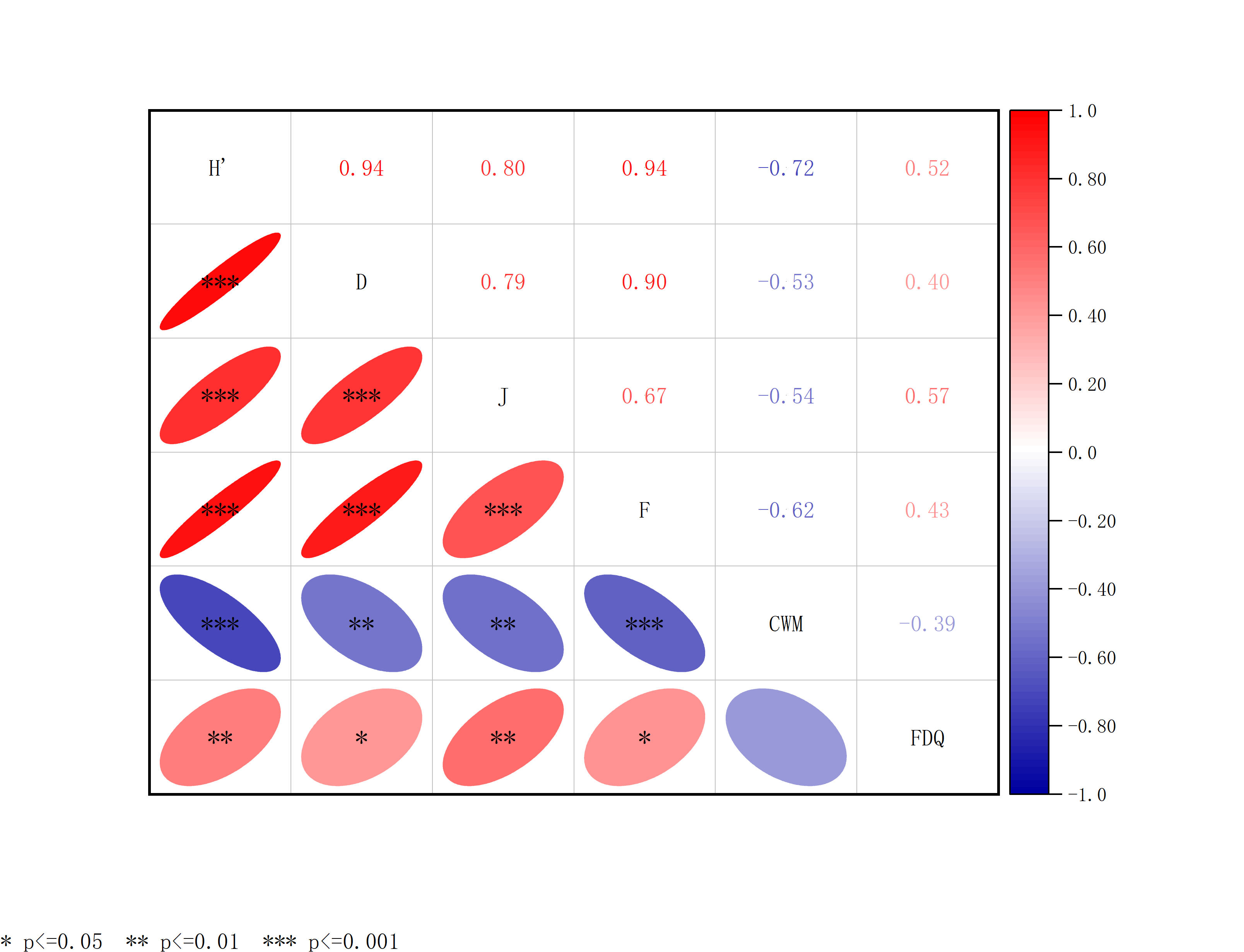
Figure 2. Relationships between species diversity and functional diversity of plant communities across *S. rostratum* invasion intensities**

* p＜=0.05 **p＜=0.01 ***p＜=0.001

Note: H': Shannon-Wiener diversity index; D: Simpson's dominance index; J: Pielou's evenness index; F: Margalef's richness index.
