# Supplementary material for: Ecological Effects of Solanum rostratum Invasion on the Diversity and Functional Traits of Native Plant Communities
Source: Ecol Evol. 2026 Jan 11;16(1):e72910. doi: 10.1002/ece3.72910 (PMC12793780; doi:10.1002/ece3.72910)
Supplement: Supplementary file 2 — Tables S1–S3: ece372910‐sup‐0002‐Tables.docx. [file ECE3-16-e72910-s002.docx]

**Table 1 Comparison of functional trait differences among native plant species under invasion by *Solanum rostratum***

|  | PH (cm) | PHa (cm) | LT (mm) | LL (cm) | LW (cm) | LA (cm²) | SWL (g) |
| --- | --- | --- | --- | --- | --- | --- | --- |
| CK | 15.037±4.152ab | 11.073±4.204 | 0.499±0.209 | 3.785±1.919a | 1.962±1.139 | 6.100±4.228a | 496.57±163.42a |
| L | 10.317±2.618a | 9.604±3.523 | 0.433±0.184 | 4.037±2.644ab | 1.920±1.477 | 8.882±6.108b | 556.53±335.01a |
| M | 10.164±5.919a | 8.270±4.671 | 0.402±0.145 | 10.631±10.885b | 2.706±2.383 | 9.960±8.702b | 463.21±253.87ab |
| H | 17.598±5.855b | 13.071±4.829 | 0.466±0.163 | 3.853±0.673a | 1.566±0.394 | 3.378±1.166a | 158.27±254.98b |
| F | 3.341 | 1.257 | 0.351 | 2.097 | 0.546 | 1.418 | 2.135 |
| P | 0.030 | 0.316 | 0.789 | 0.133 | 0.657 | 0.267 | 0.105 |

Note:Different letters on the same index indicate statistically significant differences （P < 0.05）."ns" indicates no significant difference （P > 0.05）.Abbreviations:PH: Maximum plant height （cm）；PHa: Mean plant height （cm）；LT: Leaf thickness （mm）；LL: Leaf length （cm）；LW: Leaf width （cm）；LA: Leaf area （cm²); SWL: Above-ground Biomass (g).

**Table 2 Comparison of functional trait differences among invasive plant species under *Solanum rostratum* invasion**

|  | PH (cm) | PHa (cm) | LT (mm) | LL (cm) | LW (cm) | LA (cm²) | SWL (g) |
| --- | --- | --- | --- | --- | --- | --- | --- |
| L | 36±8.165a | 26.714±10.923a | 0.459±0.084a | 5.750±0.939a | 3.168±0.794a | 8.736±2.309a | 391.492±348.49a |
| M | 50.146±5.273b | 41.480±5.118b | 0.676±0.114b | 7.911±1.662b | 3.212±0.598a | 10.284±5.026ab | 5018.51±6805.51ab |
| H | 60±15.950b | 41.746±8.218b | 0.828±0.206b | 8.399±1.687b | 5.339±0.832b | 13.770±3.035b | 8294.08±13894.55b |
| F | 8.800 | 7.082 | 11.453 | 6.285 | 17.587 | 3.132 | 3.780 |
| P | 0.002 | 0.006 | ＜0.001 | 0.009 | ＜0.001 | 0.070 | 0.044 |

**Table 3 Differences in weighted average trait values of plant communities of *Solanum rostratum spinosum* under different invasion levels**

|  | CWM_PH_ | CWM_PHa_ | CWM_LT_ | CWM_LL_ | CWM_LW_ | CWM_LA_ | CWM_SWL_ |
| --- | --- | --- | --- | --- | --- | --- | --- |
| CK | 33.648±7.744a | 19.074±4.624a | 0.331±0.112a | 2..074±0.094a | 0.757±0.282a | 1.415±0.881a | 313.68±88.848a |
| L | 23.208±6.751a | 11.281±3.041a | 0.436±0.078b | 3.174±1.166a | 1.403±0.394b | 2.693±0.508a | 126.197±29.806a |
| M | 25.303±6.185a | 17.368±4.476a | 0.608±0.129c | 5.191±0.618b | 2.260±0.367c | 6.057±1.924b | 1121.794±638.012a |
| H | 51.156±21.086a | 25.941±10.702a | 0.639±0.101c | 5.443±1.549b | 2.484±0.767c | 7.599±2.919b | 4483.033±2513.737b |
| *P* | 0.002 | 0.004 | ＜0.001 | 0.036 | ＜0.001 | ＜0.001 | ＜0.001 |
| *F* | 7.226 | 5.899 | 10.766 | 3.411 | 15.135 | 14.603 | 15.333 |

Note:CWM_PH_: Community-weighted mean trait value of maximum plant height；CWM_PHa_: Community-weighted mean trait value of mean plant height；CWM_LT_: Community-weighted mean trait value of leaf thickness；CWM_LL_: Community-weighted mean trait value of leaf length；CWM_LW_: Community-weighted mean trait value of leaf width；CWM_LA_: Community-weighted mean trait value of leaf area；Different lowercase letters indicate statistically significant differences （P < 0.05）.
